# Special Issue “Olfaction: From Genes to Behavior”

**DOI:** 10.3390/genes11060654

**Published:** 2020-06-15

**Authors:** Edgar Soria-Gómez

**Affiliations:** 1 Department of Neurosciences, University of the Basque Country UPV/EHU, 48940 Leioa, Spain; edgarjesus.soria@ehu.eus or edgar.soria@achucarro.org; 2 Achucarro Basque Center for Neuroscience, Science Park of the UPV/EHU, 48940 Leioa, Spain; 3 IKERBASQUE, Basque Foundation for Science, 48013 Bilbao, Spain

The senses dictate how the brain represents the environment, and this representation is the basis of how we act in the world. Among the five senses, olfaction is maybe the most mysterious and underestimated one, probably because a large part of the olfactory information is processed at the unconscious level in humans [1,2,3,4]. However, it is undeniable the influence of olfaction in the control of behavior and cognitive processes. Indeed, many studies demonstrate a tight relationship between olfactory perception and behavior [5]. For example, olfactory cues are determinant for partner selection [6,7], parental care [8,9], and feeding behavior [10,11,12,13], and the sense of smell can even contribute to emotional responses, cognition and mood regulation [14,15]. Accordingly, it has been shown that a malfunctioning of the olfactory system could be causally associated with the occurrence of important diseases, such as neuropsychiatric depression or feeding-related disorders [16,17]. Thus, a clear identification of the biological mechanisms involved in olfaction is key in the understanding of animal behavior in physiological and pathological conditions.

The olfactory system is a one-in-a-kind sensory system, because olfactory sensory neuro-epithelial neurons located in the nasal cavity and expressing specific odor receptor send direct projections to the main olfactory bulb (MOB), without a thalamic relay. Within the MOB, the processing of olfactory information and their relay to higher brain regions is guaranteed via a vast heterogeneity of cell-types. The work of Sanchez-Gonzalez et al. [18] defined the distribution and the phenotypic diversity of olfactory bulb interneurons from specific progenitor cells, focusing on their spatial origin, heterogeneity, and genetic profile. Fengyi Liang [19] contributes to the study of the cytoarchitecture of olfactory circuits, by reviewing the relevance of the cellular link between the olfactory receptor neurons (ORN) and the olfactory sustentacular cells (OSC). Indeed, the different olfactory functions could rely on complex cellular interactions [20], which are also regulated by neuromodulatory systems. Among them, the endocannabinoid system is emerging as a link between olfactory information and behavioral processes (e.g., memory and food intake), as reviewed here by Terral et al. [21]. Olfactory structures are the target of peripheral signals sensing the nutritional status of the organism [22], consequently affecting feeding behavior. Wu et al. [13] describe how the mitral cell (MC) activity in the MOB changes when there is a negative energy balance. Interestingly, such changes are related to impairment in olfactory discrimination. Thus, olfactory circuits represent a very interesting model system to understand general rules of information processing in the brain necessary for the species survival. In this context, several studies show that olfactory cues could also be determinant for partner selection and sexually driven behavior [2,23,24]. The work of Fraichard et al. [25] shows that the odorant-degrading enzymes (ODE) participate in mate selection. In particular, they demonstrate that the UDP-glycosyltransferase (UGT36E1) expressed in the olfactory sensory neurons (OSN) of the *Drosophila* is involved in sex pheromone discrimination. Furthermore, Liu et al. [26] present a complete review of the genetics and evolution of chemosensory detection, highlighting its potential role in modulating physiological processes, including pheromone detection. As the authors mention, chemosensitivity represents a key function in a primary common universal mechanism of eukaryote and prokaryote cells and in their interactions with the changing environment. 

Interestingly, sensing of chemical signals, in particular olfactory cues, could have a global influence at many different levels, from basic survival mechanisms to economic impacts in modern society. For example, the parasitoid wasp Ashmead, *Diachasmimorpha longicaudata* is used as a control agent in pest management to suppress fruit flies. Here, Tang et al. [27] performed a detailed transcriptome analysis showing that olfactory genes of the parasitoid wasps are expressed in response to their hosts with different scents. By using a similar methodological approach, Wang et al. [28] contribute to answering an open question about whether males and females possess the same abilities to sense odorants. Several studies have suggested that external stimuli, including courtship songs, colors and chemosensory cues, could be determinant for sex-specific behaviors. The authors reveal that, in zebrafish, chemosensory receptor genes are more expressed in males than in females, suggesting the existence of sex-specific neuronal circuits. In this sense, Tasmin L. Rymer [9] reviews the existing literature about the influence of olfactory cues in rodent paternal behavior, highlighting the role of ten genes mainly involved in aggressive responses towards intruders and pups recognition. In summary, this Special Issue reflects the state-of-the-art in olfactory research, opening new possibilities for interdisciplinary studies, from genes to behavior. 
